# Transition into the sexual and reproductive role: a qualitative exploration of Iranian married adolescent girls’ needs and experiences

**DOI:** 10.1186/s12978-021-01208-6

**Published:** 2021-07-27

**Authors:** Shahnaz Kohan, Shirin Allahverdizadeh, Ziba Farajzadegan, Morteza Ghojazadeh, Zahra Boroumandfar

**Affiliations:** 1 Nursing and Midwifery Care Research Center, Midwifery and Reproductive Health Department, Nursing and Midwifery Faculty, Isfahan University of Medical Sciences, Isfahan, Iran; 2 Student Research Center, Midwifery and Reproductive Health Department, Nursing & Midwifery Faculty, Isfahan University of Medical Sciences, Isfahan, Iran; 3 Department of Community and Preventive Medicine, Medicine Faculty, Isfahan University of Medical Sciences, Isfahan, Iran; 4 Research Center for Evidence-Based Medicine, Health Management and Safety Promotion Research Institute, Tabriz University of Medical Sciences, Tabriz, Iran

**Keywords:** Adolescent marriage, Reproductive health, Sexual issues, Qualitative study

## Abstract

**Background:**

It is necessary to invest in married adolescent girls’ health because of their roles in promoting the community and health of the next generation. Meanwhile, there are many concerns about their sexual and reproductive health. The International Conference on Population and Development emphasized the importance of access to adolescent girls to reproductive health services and counseling. In Iran, about 24% of registered marriages are to girls under 19, while their sexual and reproductive health needs have neglected. Therefore, this study aimed to identify married adolescent girls’ reproductive and sexual needs.

**Methods/design:**

Data were obtained through in-depth, semi-structured individual interviews with 36 participants, including 11 women who got married at 10 to 21 years of age, two mothers whose daughters were married in adolescence, and 23 healthcare providers and policymakers. The participants were selected through purposive sampling with maximum variation. Data collection continued until data saturation. The interviews were analyzed by qualitative content analysis. Similar codes were merged, and sub-categorization was performed, whereby similar categories were combined until the main categories that emerged.

**Results:**

The results revealed five main categories: preparing for marriage, enhancing awareness and decision—making power on sexual and reproductive health issues, developing adolescent-friendly sexual and reproductive services, providing tailored pregnancy and childbirth services, and preparing adolescents for motherhood.

**Conclusion:**

Adolescents step into marital life without the required life skills or physical and mental preparedness. They often become pregnant due to social pressures and lack of access to contraception. Therefore, in countries like Iran, where there is a high frequency of early marriage, families, education, and the health system should prepare the necessary foundation to support these adolescents and provide tailored and comprehensive sexual and reproductive health services.

## Plain English summary

Married adolescent girls have unique sexual and reproductive health needs; however, the appropriate services have not always been provided. This was a qualitative study conducted in Iran to explore married adolescent girls’ reproductive and sexual needs. We found that married adolescent girls were not prepared for marriage and had limited awareness and decision-making power on sexual and reproductive issues. There is a need to develop adolescent-friendly sexual and reproductive services. It is essential to give tailored pregnancy and childbirth services and prepare adolescents for motherhood. Adolescents step into marital life without the required life skills and physical and mental preparedness. They do not have the proper knowledge and ability to decide on sexuality and reproductive issues. Therefore, families, education, and health system should prepare the necessary foundation to support and empower these adolescents, and provide tailored and comprehensive sexual and reproductive health services.

## Introduction

Adolescence is a period of rapid physical, psychological, and cognitive changes. In the world, about one-fifth of the population is between 10 and 19-years of age, of whom 500 million live in developing countries [1, 2], and annually about 14.2 million girls marry before the age of 18 and this is expected to be at least 14% higher by 2030, nearly 15.1 million [3]. Adolescents may be under pressure or forced to marry and bear children [4].

In general, early marriage, which occurs more often in the context of poverty and gender inequality, is a barrier to educational, economic, and social opportunities [3, 5]. Globally, about 13 million girls aged 15–19 become pregnant, accounting for 11% of all births, and 95% of these births occur in low- and middle-income countries. Cultural and social factors contribute to early childbearing in developing countries. Most early childbirth occurs in the context of marriage [6]. Some adolescent girls become immediately pregnant after getting married under social and family pressures [7, 8]. For most adolescents, the pregnancy is unwanted and unplanned which is associated with health problems as well as negative physical and psychological consequences, accounting for 15% of the burden of illness and 13% of maternal mortality among adolescents [9].

Young married women often have little knowledge, education, experience, income, and power compared to adult women. Their husbands, parents, and mothers-in-law are usually the family decision-makers. They may not have the ability to refuse or resist forced sex, are unable to avoid pregnancy [10], and have restricted access to reproductive health services [11]. Studies from Nepal, Bangladesh, India, and other developing countries confirm that married adolescents’ reproductive health needs, such as preventing early and unplanned pregnancies, access to and use of contraceptives, and STI screening and treatment are not met [2, 8, 12–15]. A literature review on the health consequences of adolescent marriage in developing countries by Erulkar concluded that they were more likely to have experienced forced first marital sex, left school, and to have limited decision-making power [16]. A study conducted by Keskinoglu and others in an urban hospital in Turkey indicated the rates of pregnancy and adverse obstetric and neonatal outcomes were considerably higher in adolescent mothers [17]. According to Hosseinzadeh, becoming a mother during adolescence was difficult because they did not have enough life experience; adolescent mothers need to receive comprehensive support to meet their emotional, educational, and physical needs [18]. Safavi has also shown that married adolescent women usually enter marriage poorly equipped to negotiate adult marital roles. The lack of knowledge on sexual and reproductive health issues and early pregnancies have caused physical and psychological problems [8, 19]. Despite the high number of young married girls globally, their reproductive and sexual health needs are mostly ignored [20].

International Conference on Population and Development (ICPD) has also pronounced the special needs of adolescents, especially young women, including access to education, health, counseling services, and high-quality services for reproductive health [21]. Investing in the reproductive health needs of adolescent girls is necessary due to their dual role as a member of the community and a mother for the next generation [2].

The phenomenon of early marriage in Iranian society is deep-rooted [6]. According to Article 1041 of the Civil Law, the legal age of marriage for girls is 13 years. The marriage of girls under the age of 13 is only allowed if the girl’s parents or the court considered that the girl is ready for marring [22]. In Iran, about 24% of registered marriages are in women under 19 [23]. Adolescent marriage, early pregnancy, and childbearing is a multifaceted problem in Iran affected families, health care professionals, educators, government officials, and youth. In Iran, pregnancy among adolescents after a marriage has social approval but adverse maternal and neonatal outcomes.

Few studies in Iran have been conducted with a focus on early marriage and its implications [4, 6, 24, 25], and there is little information about adolescent sexual and reproductive health needs. This qualitative study was conducted to explore these needs and the experiences of married adolescent girls.

## Methods/design

The participants were selected by purposive sampling through eight healthcare centers where provided married teenagers reproductive and sexual health care. Researchers attended public and private healthcare facilities, introduced themselves to participants, provided a brief description of the study objectives, and invited them to participate. The place of interviews was planned according to the participants’ preference.

The inclusion criteria were a willingness to participate in the study, providing informed consent, being able to understand and express their experiences, having at least 5 years of work experience for healthcare providers including midwives, gynecologists, family physicians, reproductive health experts, psychologists, having at least 3 years of work experience for policy makers, getting married at 10 to 21 years of age for married adolescent girls and having an adolescent daughter married between the ages of 10 and 21 for the parents. Written informed consent was obtained from all participants. The data was collected through face-to-face in-depth interviews and field notes between July and November 2017. Interviews with adolescent married girls began with an open-ended question: “Please share your experience of marriage, first pregnancy, choosing a contraceptive method, deciding on the number of children…)?” and for health providers: “In your opinion, what are the needs of married teenage girls in reproductive health area?” The duration of the interviews was between 30 and 90 min, they were recorded by a digital voice recorder. Interviews continued until no new information or themes were observed in the data (data saturation). The data were analyzed using content analysis [26]. After recording each interview, the recordings were transcribed. The text was reviewed several times by the first author (SA) to achieve a comprehensive understanding of the interview. Then it was read line by line, and the important sentences and phrases were underlined, and, eventually, its essence was labeled (coding). Several initial interviews were coded at the same time by the supervisor with over 80% agreement. Similar codes were merged after the initial grouping was done, whereby similar categories were grouped together. To enhance the credibility of results, participants with maximum variation in educational level, age, age at marriage, husband’s age at marriage, and the number of children were selected. Member checks were also used, the coded interviews were returned to four participants, and agreement between the meaning ascertained by the researcher and the meaning according to participants were compared. Furthermore, a review by four qualitative experts was sought about the coding and participants’ statements. To assess transferability, a list of themes were given to people who did not take part in the study but they were similar to the participants and there were a lot of similarity between our results and their experiences.

## Results

Thirty-six individuals were interviewed: 11 girls who were married at 10 to 21 years of age residing in Tabriz, two mothers of girls who were married at 10 to 21 years of age, 11 health care providers (midwives, gynecologists, family physicians, reproductive health experts, couple therapist and psychologists), six health managers at Tabriz and Isfahan University of Medical Sciences, three policymakers from the Ministry of Health, two lawyers and one sociologist.

Fifty-five percent of the married adolescent girls had more than an 11-year age difference with their husbands. Overall, 45% of the adolescents were already mothers, and 18% of them were pregnant at the time of the interview (Table 1).

**Table 1:** Characteristics of adolescent married women

No	Current age	Age at marriage	Number of children	Education	Husband’s age at marriage
1	20	16	1	Grade 12	29
2	21	18	0	Grade 12	24
3	20	20	0	Grade 12	27
4	20	20	0	Grade 12	26
5	16	13	0	Grade 7	25
6	16	14	0	Grade 10	25
7	16	15	0	Grade 9	26
8	14	14	1	Grade 10	25
9	13	13	1	Grade 9	25
10	20	18	1	Grade 12	22
11	35	19	2	Grade 12	

The qualitative analysis yielded five main categories (Table 2).

**Table 2:** Main categories and sub-categories emerged of married adolescent girls’ experiences and sexual and reproductive needs

Main category	Sub-category
Preparing for marriage	No preparation for the marital role Lack of couple communication skills Not participating in family decision-making
Enhancing teens awareness and decision-making on sexual and reproductive issues	Inadequate premarital sexual education forced first sexual experience on wedding night Unplanned and unwanted pregnancy Lack of awareness about reproductive health care
Developing adolescent-friendly sexual and reproductive service	Inadequate access to reproductive health care services Inability of health care workers to provide appropriate services to teens Lack of counseling services for adolescents
Providing tailored pregnancy and childbirth services	Neglected prenatal care Lack of physical and psychological preparedness for childbirth
Preparing adolescents for motherhood	Attachment and bonding with their infant Inability to care for their child

### Preparation for marriage

Preparing for marriage, including the marital role, communication skills, and family decision-making emerged as the first main category.

#### No preparation for accepting the marital role

Participants showed that early marriage had disrupted their acceptance and adaptation to marital roles, despite being a child, must play the roles of a mother and a wife without the required autonomy. Also, adolescent girls were not able to take responsibility for reproductive health and to manage their social life.

*“I naturally don’t try to be curious about contraceptives,”* said a 16-year-old adolescent. “*I have not thought about pregnancy yet. Even in the counseling center, there are many words about this. I didn’t even listen to what they were saying because I can’t imagine myself as a wife”. (participant.1)*

Health services providers stated that if adolescent girls were educated about puberty and reproductive health before marriage, and trained how to manage the relationship with the opposite sex, it would help prepare them for marital life and accepting their marital role.

A 51-year-old health care workers said:

*“In a premarital education program, an adolescent should be prepared both physically and psychologically so that the adolescent will have the ability to manage her life. It is usually customary to compulsorily marry at an early age, and the person is not aware and does not know her rights. The conditions must simplify for her, and the relationship between the husband and wife should teach.”(participant.2)*

#### Lack of couple communication skills

Married adolescents discussed a lack of life skills, especially communication skills. They were unaware of how to interact with or treat their spouse and could not communicate well with other members of the new extended family. Married adolescent girls could not tolerate the judgment and advice of others about early marriage. In some cases, ineffective communication caused significant family disputes.

*“I married at the age of 16. When somebody marries at younger ages, an adolescent girl cannot understand others, doesn’t know how to behave with her husband and his family, doesn’t have any skills, and suddenly falls into a new life.” (participant.3)*

#### Not participating in family decision-making

Most adolescent girls showed very low decision-making autonomy. They expected to have greater decision-making power, so their family would consult with them on major issues, and their opinion would be respected.

A 19-year-old adolescent said:

*“My husband does whatever he wants while I can’t do anything out of his decision and comment on anything by myself.” (participant.4)*

Providers of reproductive health services were concerned about adolescents’ ability to make healthy decisions regarding health and access to health services. They stated that a lack of accessing care was related to the lack of adolescents’ participation in health decision-making.

A 57-year-old health care worker said:

*“Many problems with adolescent women are due to not knowing what decisions should make, and their husbands often have much age difference so that they cannot understand the adolescents and do not involve wives in decision-making.” (participant.5)*

### Enhancing teen’s awareness and decision-making on sexual and reproductive issues

The majority of married adolescents needed to enhance awareness and decision-making on sexual and reproductive issues. Adolescents in this study received inadequate sexual education during marriage counseling. Most of them had no preparation and were forced into the sexual relationship just after marriage, sometimes resulting in unplanned and unwanted pregnancies.

#### Inadequate premarital sexual education

Most participants stated that when they got married, they did not have insufficient information on sexual health. Because of their lack of intimacy with their mother, they learned sexual issues from friends or other sources, and after marriage, they became aware of these.

A 35-year-old participant said:

*“Although I was 17, when I got married, I did not know anything about sexual issues. I had raised in a family where my mother did not talk to us about it. I did not know what marriage is or how sexual relationship is.” (paticipant.6)*

Health services providers stated that adolescents’ experienced sexual intercourse without being prepared; they need to learn about sex before marriage.

A family physician said:

*“One of my visitors was a 13-year-old girl who suffered from post-trauma stress syndrome (PTSD) after having the first sexual intercourse on the night of her wedding. She had no idea what to do when her man approached her. So, we must first make sure that the adolescent knows the process of sexual relations.” (participant.7)*

#### Forced first sexual experience on wedding night

Many participants described their first sexual experience occurring on the first night of marital life, for which they were not ready. Most of them were stressed and afraid, and in some cases, the unwillingness to engage in sex led to disputes.

A 16-year-old adolescent said:

*“I had a lot of stress, and I was afraid of having sex early in marital life. I was very annoyed for the first time. I was scared, and I stressed so much.”(participant.3)*

The onset of sexual activity in married adolescent girls often occurs under forced conditions. Some participants stated that their first sexual experience, on the wedding night, was in the presence of family members along with the fear and shame to prove virginity.

A 35-year-old woman said:

*“On my wedding night, my mother and the aunt of my husband waited for the answer (blood on the bedsheet). They frightened me so much and said that if you did not have intercourse, people would think you were not a girl (virgin). I did not understand anything about that relationship. But I knew that I had to prove to them that I was a girl.” (participant.6)*

#### Unplanned and unwanted pregnancies

All adolescents interviewed stated that they did not understand the process of conception and did not plan for childbirth. Nevertheless, half of them were pregnant immediately after marriage under pressure from their family and society. The majority of them stated that they did not have enough knowledge about contraceptive methods and their relatives were the source of information, and sometimes they suggested contraception methods for them.

A 16-year-old adolescent said:

*“I did not want to become pregnant. However, my relatives, my husband, my mother, and my sister-in-law insisted I become pregnant. They said that if you use contraception, you may not be able to have more children. I became afraid then I had to become pregnant.”(participant.8)*

#### Lack of awareness about reproductive healthcare

Although all married adolescents started their sexual activity during the marriage, many were not aware of critical reproductive health issues and services, such as pap smears and sexually transmitted diseases (STD). The prevalence of STDs and high-risk behaviors in adolescents is higher than older women; therefore, it is essential that they have access to this information.

A 16-year-old adolescent said:

*“I got married at the age of 13. I suffered from a disease caused by sexual intercourse. I had no problem before marriage. I did not do cervical sampling until now, because I am afraid I go there and get annoyed.” (participant.3)*

A health service provider with 26 years of work experience said:

*“STDs are more prevalent in adolescents, and adolescent sexual behaviors are different as well. For example, they may use alcohol or drug, have risky sexual behaviors, or they may become infected by various types of STDs, while they are not aware of the consequences.” (participant.9)*

### Developing adolescent-friendly sexual and reproductive service

Participants described adolescents’ poor access to appropriate reproductive health services. Adolescents and providers also stated that health care workers should provide reproductive services and counseling for adolescents.

#### Inadequate access to reproductive healthcare services

Most adolescents talked about the fact that their spouses and their families did not allow them to go to healthcare centers alone.

A 19-year-old adolescent said:

*“I usually go to the doctor's office with my husband. If my husband were at work, I would go with my mother-in-law. If no one could come with me, I was not able to go to the doctor even if I had a problem.” (participant.10)*

The view of most providers was that adolescents did not have reasonable access to reproductive healthcare services. They stated that financial, cultural, and social issues prevented adolescents’ access to healthcare centers.

A healthcare provider said:

*“The husband of one of my adolescent visitors was very strict and did not allow her to go to a doctor during pregnancy. She was told to just go to the healthcare center and do whatever they say.” (participant.11)*

#### Inability of health care workers to provide appropriate services to teens

Many teens expressed that they felt embarrassed about providers’ judgments about their early marriage/pregnancy. They discouraged married adolescent girls from seeking services and from attending clinics or follow-up visits. They wished health workers would demonstrate more respect and provide health information specific to their age and level of knowledge.

An adolescent married at age 15 said:

*“The staff of the reproductive healthcare center should well know adolescents, know the behaviors, understand them, and appropriately talk to them.” (participant.12)*

Most health care workers stated that they did not have specific training in communication or providing services to adolescents and that they did not have enough time to focus on adolescent problems.

A 42-year-old provider said:

*“To improve the reproductive health of adolescents, health care workers need to train in communication with them and should take specific courses for providing services to adolescents.” (participant.13)*

#### Lack of counseling services for adolescents

Most providers talked about the need for comprehensive adolescent counseling during the marriage as well as counseling with the couple therapist and psychologist at the time of marriage.

A policymaker said:

*“Candidate adolescents for marriage need a lot of training, such as couple counseling, which is mentally more effective for adolescents.” (partcipant.14)*

Some health services providers also mentioned the legal dimensions of adolescent marriage and stated that it would be better to address this during marriage counseling.

The head of a marriage registration office said:

*“Legal issues and reproductive rights should be propounded during adolescent marriage and included in marriage counseling, while counseling provided is not potent. Indeed, there should be sophisticated counselors and health system to invite parents and daughters and explain these matters to them.” (participant.15)*

Providers emphasized that psychological counseling should be included in marriage counseling for adolescents because psychological problems may occur during the early marriage for adolescents, and there may be serious psychiatric problems such as depression.

A doctor said:

*"I have seen many cases that adolescents had severe mental problems at the beginning of marital life and even before the marriage which required psychotherapy." (participant.16)*

Many health care workers believed that adolescents needed continuous counseling to improve marital skills and plan for their pregnancy.

A policymaker said:

"*Counseling during the marriage is compulsory, and it is a bottleneck in identifying adolescents, for them, we should have a continuous plan after marriage." (participant.17*)

### Providing tailored pregnancy and childbirth services

Inadequate pregnancy-specific services and a lack of physical and mental readiness for childbirth is a concern among newly married adolescent girls.

#### Neglected prenatal care

The majority of health care providers stated that prenatal care for adolescents was routinely performed based on national maternal care guidelines; however, the specific needs of adolescents were not considered. Adolescent girls were placed in a high-risk group only because of their low age, so health care workers should be more sensitive to their needs and responsible for their follow-up. Health Services providers state that separating the care of pregnant adolescents from adult pregnant women requires more time and cost, which is not feasible in the current healthcare system.

The head of population and family health group in the health department of Tabriz University of medical science said:

*“Adolescent mothers do not separate from other adult women … It is not possible to separate the processes of prenatal caring of married adolescents. Because we have not any space and cost for them.” (participant.18)*

Adolescents pointed out that health care workers should support them to cope with pregnancy by teaching them the danger signs of pregnancy and common complaints, providing psychological support, and advising of proper nutrition.

A participant said:

*“In the last days of my pregnancy, I was 48 kilos, while I was 57 kilos before pregnancy. I could not eat well in the early days, so I lost weight. My body became weak, and I could not sleep well and do not have adequate food to be supplied. The doctor also did not pay attention and did not understand me.”(participant.6).*

Most adolescents stated that there was no adolescent-specific pregnancy care, and some of them did not attend antenatal care. Most mentioned that the disrespectful behavior of health care workers discouraged them from attending prenatal care.

A 16-year-old adolescent said:

*"I got pregnant at age 14, and when I attended the prenatal care center, their behaviors discouraged me from attending follow-up visits." (participant.19)*

#### Lack of physical and psychological preparedness for childbirth

Health services providers stated that delivery is difficult for adolescents, and many suffer psychological trauma and may have a difficult delivery due to poor physical fitness. Health care workers stated that adolescents suffer from a great deal of fear during childbirth and cannot bear labor pain.

A 48-year-old midwife said:

*"A 16-year-old adolescent who had her second childbirth, she was moaning a lot and cried out that I do not want this baby; we had to calm her pain with pethidine." (participant.20)*

Adolescents stated that they did not understand the delivery process or have sufficient information about the onset of labor. This lack of awareness jeopardizes both their health and the health of the baby.

A 19-year-old adolescent said:

*“I had the water break. I was embarrassed to tell my mother-in-law. We went to the doctor, and she said that it is the time of delivery. I had no pain at first. I went to the hospital immediately to get hospitalized. I have injected delivery serum because I did not have pain, and the pain became severe, I could not breathe, and they gave me oxygen. I felt strangled. When I was there, they said that if it goes like this we should take you to the operation room, and I was afraid of operation…..”(participant.21)*

### Preparing adolescents for motherhood

The fifth main category was preparing adolescents for motherhood. Most mothers described difficulties with attachment to their unborn babies and infants. They also felt unable to nurture and take care of their baby because they were ill-prepared.

#### Attachment disorder with fetus and baby

Most of the providers stated concern regarding the attachment of adolescent mothers with their babies. It was felt that this lack of bonding was related to being unprepared or the result of an unwanted pregnancy. As a result, adolescent mothers often conceal and reject their pregnancies. This behavior and feeling may result in abandonment, neglect, and the absence of bonding with their child at birth, and the adolescent’s family tended to take care of the baby, so the adolescent mother lost the opportunity to bond with the baby.

A midwife in the delivery room said:

*“An adolescent mother did not intend to hug the infant after childbirth; she did not feel the mothering love. She said that I could not accept the child. Because I oppressed, I married soon, and I do not have that mothering feeling towards the child.”(participant.20)*

In this study, half of the adolescents gave birth a short time after the marriage, and most were ambivalent about the child. Furthermore, due to extensive family support provided, there was little opportunity for the mother to create an emotional bond with the child.

A 35-year-old participant said:

*“I gave birth to my daughter at age 19. I did not know how to spend time with the child, play with my daughter and communicate with her. I could not even hug or kiss my daughter. I was embarrassed.”(participant.6)*

#### Inability to raise and take care of the child

The viewpoint of most adolescents was that they required more education about how to care for their babies. Some of them had children at the age of 15–16 and felt unskilled and emotionally enable for child care; therefore, the adolescent’s family took care of the child.

A 16-year-old adolescent said:

*“I married at age 13. I was concerned that I cannot take care and raise my child properly because I am still a child.” (participant.22)*

According to health services providers, many adolescent mothers did not have the skills to care for and feed their children, housekeeping, or even cooking, which makes it difficult for them to be a mother. They also stated that they did not know much about the signs of childhood illness and that they did not know how to care for a child if he or she became ill.

A reproductive health expert said:

*“The child of an adolescent mother had a fever due to cold. The mother not only did not notice the child's fever but also developed a seizure fever. The mother's inability caused the baby's seizures to continue with high severity and to take many medications to control the seizures.”(participant.23)*

## Discussion

This research identified five main categories related to married adolescent girls’ experiences and needs during the transition into a sexual and reproductive role. These themes included the need to prepare for marriage, enhancing awareness and decision-making power on sexual and reproductive issues, developing adolescent-friendly services, providing tailored maternity services, and preparing adolescents for motherhood.

The view of the majority of participants described a lack of preparedness for the martial role, including the need for communication skills. Similar findings by Hamid et al. showed that all married adolescent girls need to prepare before marriage and be aware of couple communication [27]. Erulkar found that adolescent girls married under the age of 20 should acquire skills such as marital skills [16]. According to the view of adolescents who participated in our study, they did not engage in decision making. Shahabuddin also reported that adolescent women have less decision-making power, as well as little knowledge about the role of the mother and wife [28]. From the viewpoint of Santhya and Jejeebhoy, the age of marriage is an important factor in the autonomy and decision-making power of marital life. The younger adolescent, the less her decision-making power [29]. Simbar and Alizadeh reported that educating adolescents about reproductive health issues can help them to develop life skills such as communication, and negotiation so that this transition from childhood to adulthood can complete [30].

Based on the findings of this study, enhancing awareness and decision-making power on sexual and reproductive issues was one of the identified needs. There is no convenient sex education program for adolescent girls during the marriage, and often adolescents begin sexual relationships right after marriage to prove virginity. Montazeri et al. indicated that girls could not be aware of sexual issues considering their age and given limited access to reliable knowledge [4]. According to Erulkar et al., sexual intercourse is compulsory at the beginning of the marriage, and even married adolescent girls reported the experience of intimate person sexual violence [16]. In a study by Klingberg-Allvin et al., female adolescents were forced to satisfy their husbands sexually based on their husband’s desires [31]. A similar study in Iran showed that after marriage, most adolescent married girls felt, early marriage has led to unpleasant or coercive sexual experiences for some of them [32]. The results of Santhya’s study suggested that forced sex of newly married adolescent girls is common. Adolescent girls are completely unprepared to have sex with their spouses, and their first sexual contact is traumatic, unpleasant, and painful. Adolescents also were completely unaware of sexual and reproductive health and had low decision-making power and negotiating skills on sexual matters [33]. The findings of Bostani’s study showed that as long as a healthy and satisfying sexual relationship between the couples is one of the most crucial components of cohabitation, sexual health education is essential for young couples about to get married [34].

Our results indicated that the majority of married female adolescents were under family pressure for childbearing after marriage, half of them experienced unplanned and unwanted pregnancies and had insufficient awareness of contraceptive methods. The results of a survey in Pakistan showed that the mother-in-law or the head of the household was taking all decisions about using contraceptives by a newly married adolescent daughter-in-law [27] Chandra-Mouli et al. indicated that adolescent girls are pressured to bear a child after marriage. They have little information about the variety of contraceptive methods, and they did not use them because of the fear of its complications on fertility in the future [35]. From Blum’s perspective, newly married adolescent women in developing countries faced cultural expectations and social pressures and had to prove their fertility immediately after marriage through the pregnancy [36].

The necessity of developing adolescent-friendly sexual and reproductive health services was another need that the majority of the participants emphasized in this study. From the perspective of adolescents and providers, reproductive health services are not appropriate to meet adolescent needs and desires. Adolescents do not seem to have enough access to reproductive health services, and they are unaware of most of their reproductive health needs, such as the need for pap smears and STD screening. According to Simbar’s study in Iran, adolescents need reproductive health services that are settled for them and meet their needs, which should provide in the form of adolescent-friendly services [30]. Edmeades also suggested that promoting adolescent reproductive health, identifying married adolescents’ reproductive health needs, separating their services from other public healthcare services, and developing adolescent-friendly reproductive health services are necessary for Ethiopia [37].

Providing tailored care in pregnancy and childbirth was another requirement emphasized by health services providers in this research. Adolescent mothers received insufficient services and did not have physical and psychological preparedness for childbearing. Defo’s study showed that young mothers suffer from complications due to physical immaturity and lack of psychological preparation during pregnancy and childbirth, at times causing physical disability [10]. Also, a viewpoint of a study in Uganda young women stated some health care workers had a ‘don’t care attitude’ and that they were sometimes rude and abusive to patients. These practices tend to de-motivate adolescents from utilizing public health units [9].

The findings of the present study suggested that married young women need to be prepared for maternal roles. Due to childbirth at an early age, most of the adolescents experienced disturbances in their attachment to the fetus and the baby, and felt an inability to take care of the child. According to Salehi’s systematic review in Iran, effective strategies to strengthen the maternal-infant attachment includes a planned and wanted pregnancy, maternal awareness about pregnancy and fetus development, number of prenatal and postnatal visits, and family supports during pregnancy [38]. A similar finding by Klingberg-Allvin et al. revealed that adolescent mothers felt severe problems and difficulties in caring for their children in Vietnam [31]. According to Hackett et al., adolescent girls realized that childbearing at a young age was an obstacle to the best practice child-raising because they were ready neither physically nor psychologically to take care of the child in Bangladesh [39].

## Limitations

Generalization of the findings in the present study, considering its qualitative approach, should be done cautiously. Although qualitative studies are not designed for the generalization of the results, they are useful for those who are willing to use the results while considering the limitations. By selecting participants with maximum variation, seeking the guidance and supervision of experts, and the use of external reviews, the accuracy, and transferability of the data can be increased.

We could persuade just two adolescent mothers to participate in interviews, and we were unable to involve adolescent’s husbands. In total, we were able to interview only 11 adolescents, which prevented us from the further generalization of these results.

## Conclusion

Adolescent girls enter marital life with little or no skills and physical and mental preparedness for spousal and maternal roles. Therefore, from marriage to pregnancy and childbirth, there is a need for special services such as raising awareness of sexual relationships, planned pregnancy, access to reproductive health services, and the preparedness for pregnancy, childbirth, and training for child care. Therefore, all married adolescents should receive comprehensive care from a competent and well-informed health team and health centers with tailored services for adolescents.

There is a need to raise awareness among girls, parents, teachers, and community leaders. There is also a need to hold the government accountable for enforcing the legal age of marriage for girls. Further programs to enhance married girls’ autonomy within their marital life and those that encourage education and generate livelihood opportunities need to be simultaneously developed.

## Data Availability

This paper is based on qualitative research and further information about the data is available from the corresponding author on request.

## Abbreviations

SRH: Sexual and reproductive health
MDGs: Millennium Development Goals
ICPD: International Conference on Population and Development
PTSD: Post-traumatic stress disorder
STDs: Sexually transmitted diseases
SDGs: The Sustainable Development Goals
